# Criptococosis diseminada por terapia biológica, se debe gestionar el riesgo.

**DOI:** 10.7705/biomedica.6239

**Published:** 2022-06-01

**Authors:** Efraín Guillermo Sánchez, David Acosta, Juan Álvarez, Gabriela Sánchez, Julio García-Casallas

**Affiliations:** 1 Departamento de Medicina Interna, Fundación Cardioinfantil - Universidad del Rosario, Bogotá, D. C., Colombia Universidad del Rosario Universidad del Rosario Bogotá, D. C. Colombia; 2 Clínica Universidad de La Sabana, Universidad de La Sabana, Chía, Colombia Universidad de la Sabana Universidad de La Sabana Chía Colombia; 3 Grupo de investigación Evidencia Terapéutica, Facultad de Medicina, Universidad de La Sabana, Chía, Colombia Universidad de la Sabana Universidad de La Sabana Chía Colombia

**Keywords:** Cryptococcus neoformans, criptococosis, terapia biológica, errores de medicación, Cryptococcus neoformans, cryptococcosis, biological therapy, medication errors

## Abstract

**Introducción.:**

Se han descrito múltiples efectos adversos con el uso de la terapia biológica para enfermedades autoinmunitarias, muchos de ellos secundarios al estado de inmunosupresión, como las infecciones bacterianas, fúngicas o virales.

**Caso clínico.:**

Se presenta el caso de una mujer de 64 años con diagnóstico comprobado de criptococosis diseminada secundaria al uso de tofacitinib. Se descartaron otras causas de inmunosupresión, como infección por el virus de la inmunodeficiencia humana (HIV). Tres años antes se le había diagnosticado artritis reumatoide y se encontraba en tratamiento farmacológico con un agente biológico que inhibe las enzimas JAK. Se han descrito muy pocos casos de criptococosis pulmonar y meníngea en este tipo de pacientes.

**Conclusión.:**

Este reporte de caso es útil para que otros médicos tratantes tengan presente la posibilidad de este tipo de infección fúngica invasora asociada con la terapia biológica y el enfoque de gestión de riesgo.

## Abstract (en)

**Introduction:**

Multiple adverse effects have been described for the biological therapy in autoimmune diseases including many secondary to immunosuppression producing bacterial, fungal, or viral infections.

**Clinical case:**

We present the case of a 64-year-old female patient with proven disseminated cryptococcosis secondary to the use of tofacitinib. Other possible causes of immunosuppression such as the human immunodeficiency virus (HIV) were ruled out. The patient had been in treatment for rheumatoid arthritis diagnosed three years before. This drug is a biological agent that inhibits JAK enzymes. Very few cases of pulmonary and meningeal cryptococcosis in this type of patient have been described in the literature.

**Conclusion:**

This case report should be useful for other clinicians to bear in mind the possibility of this type of invasive fungal infection associated with biological therapy and to take a risk-management approach.

Los medicamentos de origen biológico han desplazado a los de origen sintético a medida que se ha ampliado el conocimiento fisiopatológico y molecular de las enfermedades no transmisibles y transmisibles; diversas especialidades se han beneficiado de ellos, lográndose un buen control de la enfermedad y, en algunos casos, la disminución de la mortalidad. En el caso de la enfermedad reumática, varios medicamentos biológicos han demostrado un mejor control de la progresión de la enfermedad y menos efectos secundarios. Sin embargo, estos deben vigilarse y, sobre todo, manejarse adecuadamente ^1^.

## Caso clínico

Se presenta el caso de una paciente de 64 años que ingresó al servicio de urgencias por una caída desde su propia altura que le ocasionó un trauma craneoencefálico.

**Cuadro 1:** Exámenes de laboratorio básicos de la paciente en el momento del ingreso

Examen	Valor o porcentaje
Leucocitos	11.590 células/mm^3^
Linfocitos	530 células/mm^3^
Neutrófilos	10.800 células/mm^3^
Hematocrito	41 %
Hemoglobina	15 mg/dl
Nitrógeno ureico	7,3 mg/dl
Creatinina	0,7 mg/dl
Glucemia	106 mg//dl
Bilirrubina total	0,5 mg/dl
Fosfatasa alcalina	45 U/L
Transaminasa pirúvica	11,7 U/L
Transaminasa glutámica	21,2 U/L

**Figura 1:**
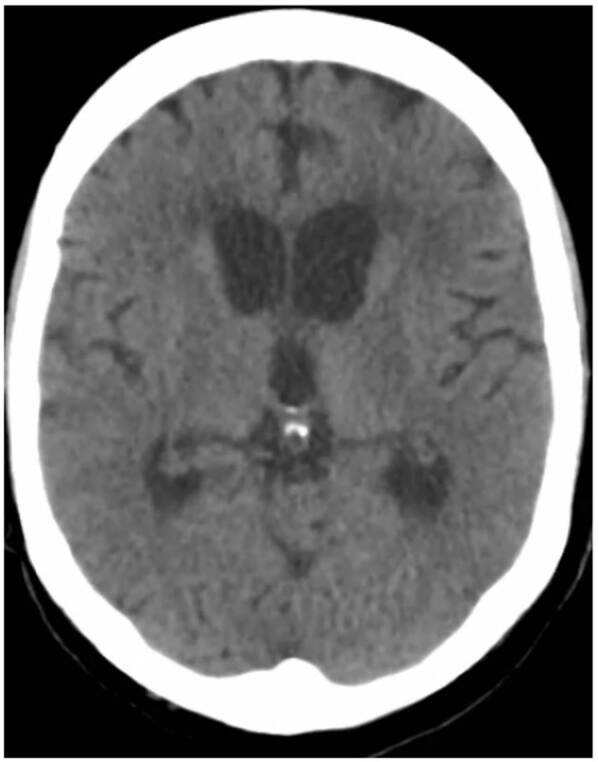
Tomografía de cráneo que muestra aumento de tamaño de los ventrículos laterales y el tercer ventrículo, indicativo de hidrocefalia no comunicante

Tenía antecedentes de hipertensión arterial, hipotiroidismo y artritis reumatoide que venía siendo tratada con 5 mg de prednisolona al día y 5 mg de tofacitinib cada 12 horas desde hacía tres años. No había informado de efectos adversos asociados con el medicamento.

En el ingreso, no se reportó ninguna alteración estructural en la tomografía (TC) de cráneo. A pesar de no presentar síntomas urinarios, se solicitó un uroanálisis cuyo resultado sugería infección, por lo que se inició la administración de cefepime y se solicitó el urocultivo; además, por ser una paciente con inmunosupresión, se solicitaron hemocultivos.

Los exámenes básicos de laboratorio hechos en el momento del ingreso se resumen en el cuadro 1. En ellos, llama la atención la leucocitosis y la linfopenia relativas de la paciente. Los hemocultivos por MALDI-TOF revelaron la presencia de *Cryptococcus neoformans*.

**Figura 2:**
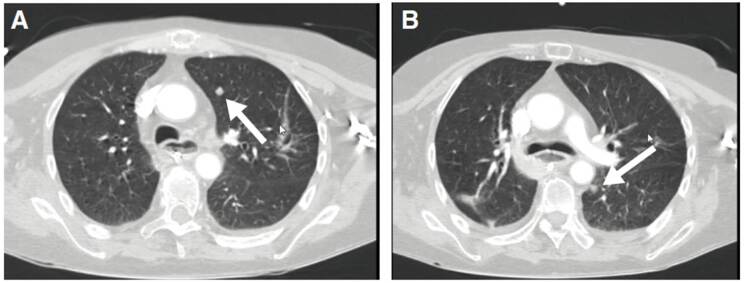
A. Nódulo con densidad de tejidos blandos en el lóbulo superior del pulmón izquierdo. B. Nódulo con densidad de tejidos blandos en el lóbulo inferior del pulmón izquierdo

En la segunda TC de cráneo se encontró hidrocefalia no comunicante (figura 1). En la punción lumbar, se obtuvo un líquido raquídeo de aspecto turbio, cuya viscosidad no permitió medir la presión de apertura; las proteínas estaban en 1.000 mg/dl, los leucocitos en 2 por mm^3^, la glucosa en 33 mg/dl y el antígeno de *Cryptococcus* dio positivo. En el cultivo del líquido cefalorraquídeo, se detectó *C. neoformans*. Además, una tomografía de tórax de alta resolución demostró compromiso nodular multilobar (figura 2).

Con estos resultados, se diagnosticó meningitis aguda por *C. neoformans* y criptococosis diseminada. Se inició el tratamiento con anfotericina B liposómica en dosis diarias de 5 mg/kg y fluconazol, 400 mg/día, dado que no se disponía de 5-flucitosina. A pesar del tratamiento instaurado, la condición clínica de la paciente empeoró y, finalmente, falleció.

## Consideraciones éticas

La paciente dio el consentimiento para hacer público su caso y las imágenes de diagnóstico. La publicación del caso fue aprobada por el comité ético institucional.

## Discusión

La criptococosis es una enfermedad fúngica invasiva producida por las especies *neoformans* y *gatti* del hongo del género *Cryptococcus*, distribuidas a nivel mundial ^2^. Los factores de riesgo descritos para contraerla son: infección por HIV, tratamiento con esteroides, trasplante de órgano sólido, neoplasias hematológicas, lupus eritematoso sistémico, artritis reumatoide, sarcoidosis, anticuerpos monoclonales (etanercept, infliximab, alemtuzumab), diabetes mellitus, falla renal y enfermedad hepática crónica ^2^.

El diagnóstico se basa en la localización de la infección. Cuando esta se ubica en el sistema nervioso central, puede causar cefalea, fiebre, neuropatías craneales, alteración de la conciencia, letargia, pérdida de la memoria y signos de irritación meníngea.

En cuanto a las imágenes, la TC de cráneo puede evidenciar hidrocefalia, nódulos múltiples, masa que ocupa espacio, aunque también puede no revelar ninguna anormalidad.

En la punción lumbar, se puede encontrar una presión de apertura de más de 20 cm de agua en el 50 % de los casos, con leucocitos usualmente de menos de 50/mm^3^ y de predominio linfocitario, glucosa entre normal y baja, y proteínas de más de 50 mg/dl. La tinción con tinta china fue positiva en el 50 % de los casos sin presencia de HIV y, en el 85 % de los casos, con el virus. El antígeno en la prueba de látex en líquido cefalorraquídeo tiene una sensibilidad cercana al 100 % y una especificidad mayor de 85 %, y revela la reproducción activa del hongo. En el cultivo de líquido espinal, la sensibilidad es cercana al 85 % y, en hemocultivos, al 70 % ^3^.

El tofacitinib es un medicamento oral que actúa inhibiendo las enzimas JAK (*Janus Kinase*), por lo cual disminuye el número de células asesinas naturales (*Natural Killer*, NK) y los valores de inmunoglobulinas G, M y A, así como el de la proteína C reactiva. Tiene buena absorción oral, con biodisponibilidad del 70 % que se puede reducir al 30 % si se ingieren alimentos grasos. Su unión a proteínas es de alrededor del 50 %, el volumen de distribución es de 87 litros, su vida media es de tres horas en tableta de liberación inmediata y, de 6 a 8 horas, en las de liberación prolongada. El 70 % se metaboliza en el hígado, sobre todo mediante el citocromo CYP450- 3A4, y el 30 % se excreta en la orina.

El tofacitinib está indicado en el tratamiento de artritis reumatoide, artritis psoriásica, artritis juvenil y colitis ulcerativa ^4^. Se han descrito efectos adversos serios, como la muerte súbita o el linfoma, y otros, como infecciones y nasofaringitis (>10 %), hipertensión arterial sistémica, cefalea, erupción cutánea, acné, dislipidemia, infección urinaria, anemia, falla renal, fiebre, toxicidad medular, pancitopenia, prolongación del intervalo QR, perforación gastrointestinal, hepatotoxicidad y tromboembolia (1-10 %) ^1^.

En cuanto a las infecciones asociadas con la terapia, se ha descrito una incidencia anual del 43 %. Las infecciones más serias son neumonía, herpes zóster diseminado, infección urinaria, celulitis, gastroenteritis y diverticulitis ^1^. Los factores de riesgo reportados son: uso adicional de corticoides, un puntaje que indique gravedad de la enfermedad, linfopenia de menos de 500 células/mm^3^, tener más de 65 años y ser de procedencia asiática ^4^.

La infección por herpes zóster parece depender de la dosis (mayor con dosis de 10 mg). En la mayoría de los casos, se afectan uno o dos dermatomas según lo reportado en el estudio OCTAVE, cuyo breve seguimiento impidió la detección de efectos adversos adicionales ^5^. La incidencia fue de 3,9/100 pacientes al año en un seguimiento de 6.194 pacientes en tratamiento con una dosis de tofacitinib de 5 o 10 mg cada 12 horas. Además, con el tofacitinib, se ha informado un cociente de riesgo de 2 para la infección por herpes zóster, en comparación con otros productos biológicos. La infección por herpes zóster se maneja con un antiviral y la suspensión del producto biológico, y se obtiene mejoría terapéutica, en promedio, a los 21 días ^6^.

También, se ha descrito reactivación de la tuberculosis y otras infecciones por micobacterias. En una evaluación de 5.671 pacientes con antecedente de artritis reumatoide en tratamiento con tofacitinib, se encontraron 60 casos de infecciones oportunistas por tofacitinib. Se reportó una incidencia de 0,2 por 100 pacientes al año de tuberculosis. Antes de administrar el medicamento, debe descartarse una tuberculosis latente o activa, para lo cual parece ser mejor la prueba de tuberculina combinada con pruebas de liberación de interferón gamma, sobre todo para el diagnóstico de la tuberculosis latente. Este tamizaje debe hacerse en sitios con endemismo alto o intermedio, cada tres meses durante seis meses y, luego, cada 18 meses ^7^. Además, en el manejo de la tuberculosis latente o activa debe tenerse en cuenta que la rifampicina disminuye al 85 % la biodisponibilidad del tofacitinib, por lo que debe tratarse por lo menos un mes antes de iniciar la administración del producto biológico ^7^. Los casos de tuberculosis pueden ser pulmonares o extrapulmonares y la mayoría se presentan en sitios con alta prevalencia de la enfermedad. No se registró aumento del riesgo con el uso de corticoides ^6^.

En los estudios clínicos con tofacitinib se excluyeron los pacientes con hepatitis B crónica o hepatitis C. En un estudio en Taiwán de 116 pacientes tratados con tofacitinib que tenían antecedente de hepatitis B (6 portadores y 75 con infección resuelta), se encontró que en el 1,7 % de la población total esta se reactivó. Por ello, los pacientes con antígeno de superficie o anti-HBc total positivo deben recibir terapia antiviral profiláctica durante el uso del producto biológico ^6^.

En el estudio ya comentado realizado en más de 5.000 pacientes, se encontró una incidencia de 0,21 por 100 pacientes/año de infecciones oportunistas diferentes a la tuberculosis, lo que aumentó con el uso de esteroides. Entre las infecciones detectadas, nueve fueron por *Candida*, cuatro por *Pneumocystis* y tres por *Cryptococcus*^7^.

Algunos reportes de casos documentan infecciones fúngicas por *Cryptococcus* o histoplasma. Entre estos se cita el caso de una paciente de 65 años que recibió tofacitinib durante seis meses con resultados efectivos para la enfermedad de base (psoriasis), pero que tuvo que ser ingresada en varias ocasiones al servicio de urgencias por síntomas respiratorios hasta que se hizo el diagnóstico de infección por *Cryptococcus* al dar positivo el antígeno en suero ^8^.

Por último, en un estudio publicado en el 2013 con 792 pacientes con artritis reumatoide, se reportó que uno de 391 pacientes en el grupo tratado con 10 mg de tofacitinib tuvo neumonía por *Cryptococcus* a los seis meses de administración del medicamento ^9^b. La escasa información de la asociación del tofacitinib y las infecciones por *Cryptococcus* refuerza la necesidad de reportar los casos que se presenten en la práctica clínica.

Dado que el medicamento disminuye la inmunogenicidad de las vacunas en adultos, se recomienda mantener actualizado el esquema de vacunación (en especial contra herpes zóster) ^1^. No se recomienda administrar vacunas vivas atenuadas cuando se está administrando tofacitinib ^10^.

El caso que reportamos tuvo la particularidad de un compromiso diseminado de la infección por *C. neoformans*, algo que no se ha reportado previamente asociado con el uso de tofacitinib. Cabe recalcar que la paciente tenía dos factores de riesgo adicionales: el uso de esteroides (aunque en dosis bajas) y la artritis reumatoide controlada con el producto biológico.

## Conclusión

El tratamiento con productos biológicos puede dar lugar a infecciones asociadas con su mecanismo de acción, con una mayor incidencia en pacientes que tienen factores de riesgo. El médico tratante debe gestionar el riesgo a partir del conocimiento derivado de las pruebas clínicas y de los reportes de la literatura especializada, con el fin de aumentar la seguridad de los pacientes que se benefician de estos medicamentos.
